# Histone lactylation promotes cell proliferation, migration and invasion through targeting HMGB1 in endometriosis

**DOI:** 10.7555/JBR.37.20230095

**Published:** 2023-11-15

**Authors:** Jie Chen, Pengfei Qin, Yanli Sun, Suping Han

**Affiliations:** 1 Department of Obstetrics and Gynecology, Women's Hospital of Nanjing Medical University, Nanjing Maternity and Child Health Care Hospital, Nanjing, Jiangsu 210004, China; 2 Department of Gynecology, the First Affiliated Hospital of Nanjing Medical University, Nanjing, Jiangsu 210004, China

**Keywords:** endometriosis, ESCs, lactate, lactylation, HMGB1

## Abstract

Endometriosis is defined as a condition with endometrium-like tissues migrating outside of the pelvic cavity. However, the mechanism of endometriosis is still unclear. Lactate can be covalently modified to lysine residues of histones and other proteins, which is called lactylation. The results showed that the higher level of lactate and lactate dehydrogenase A enhanced the histone H3 lysine 18 lactylation (H3K18lac) in ectopic endometrial tissues and ectopic endometrial stromal cells than that in normal endometrial tissues and normal endometrial stromal cells. Lactate promoted cell proliferation, migration, and invasion in endometriosis. Mechanistically, lactate induced H3K18lac to promote the expression of high-mobility group box 1 (HMGB1) in endometriosis, and HMGB1 knockdown significantly reduced the cell proliferation, migration, and invasion of the lactate-treated cells through the phosphorylation of AKT. In conclusion, lactate could induce histone lactylation to promote endometriosis progression by upregulating the expression of HMGB1, which may provide a novel target for the prevention and treatment of endometriosis.

## Introduction

Endometriosis is a gynecological disease that is characterized by the endometrium exists outside the normal location^[1]^. All around the world, an estimated 10% of women are in their reproductive age^[2–3]^. Although endometriosis has been defined for almost a century^[4]^, the treatment of endometriosis is still limited due to unclear mechanisms. Thus, it is urgent to uncover the mechanism of endometriosis to provide a novel target for endometriosis.

Lactate is one of metabolites during the process of glycolysis^[5]^. Lactate can mediate the process of post-translational medication in histone lysine residues, named lactylation^[6]^. In tumor-infiltrating myeloid cells, histone lactylation enhances the expression of methyltransferase like 3 (METTL3) and promotes the immunosuppressive capacity^[7]^. In hepatocellular carcinoma (HCC), the residue lysin 28 lactylation impedes adenylate kinase 2 (AK2) activity to induce the proliferation and metastasis of HCC cells^[8]^. More and more proteins have been identified to be lactylated. For instance, one study showed that snail1 lactylation was induced by lactate and promoted endothelial-to-mesenchymal transition after myocardial infarction^[9]^. Several studies demonstrated that the levels of lactate were increased in follicular fluids of the patients with endometriosis, which was associated with endometriosis-associated symptoms^[10–11]^. However, the role of lactylation in endometriosis have not yet been investigated.

High-mobility group box 1 (HMGB1) is important in cell replication and differentiation as well as gene transcription and repair^[12]^. The HMGB1 expression is closely correlated with cancer-invasive and metastatic activity in HCC^[13]^, prostate cancer^[14]^, glioma^[15]^, and nonsmall-cell lung cancer^[16]^. The receptor for advanced glycation end products (RAGE)-HMGB1 interaction could activate MAPKs and NF-κB pathways to promote tumor growth^[17]^. Furthermore, lactate dehydrogenase A (LDHA) could regulate the expression of HMGB1 by histone lactylation in cerebral ischemia/reperfusion injury^[18]^. In the present study, we demonstrated that the lactate could induce histone lactylation to promote endometriosis progression by upregulating the expression of HMGB1, which may provide a novel target for the prevention and treatment of endometriosis.

## Materials and methods

### Subjects and tissue collection

The endometrial tissues were collected from women of reproductive age with endometriosis from Nanjing Maternity and Child Health Care Hospital between January 2021 and December 2021. An informed consent was obtained from each participating woman. The study procedures were approved by the Medical Ethics Committee of Nanjing Maternity and Child Health Care Hospital and carried out following the ethical standards of the Helsinki Declaration (NO. [2020]KY-037). Inclusion criteria: women in reproductive-age, normal hepatic and renal function, and diagnosed with endometriosis of a surgical indication. Exclusion criteria: women with ongoing pregnancy, with current hormone treatment, and diagnosed with cancers. All endometrial samples were collected under sterile conditions and stored in liquid nitrogen. Normal endometrial tissues were collected from outpatient hysteroscopy. The baseline information of patients is summarized in ***Table 1***.

**Table 1:** Baseline characteristics in two groups

Baseline characteristics	Control group (*n=*10)	Endometriosis group (*n=*10)
Age (years, mean±SD)	34.65±3.65	29.21±6.98
BMI (kg/m^2^, mean±SD)	23.87±4.36	21.98±2.55
Previous hormone treatment [*n* (%)]	2 (20.0)	1 (10.0)
Desire for pregnancy [*n* (%)]	7 (70.0)	8 (80.0)
History of infertility [*n* (%)]	3 (30.0)	1 (10.0)
Abbreviations: SD, standard deviation; BMI, body mass index.		

### Cell isolation and culture

The isolation and culture of ectopic endometrial stromal cells (eESCs) and normal endometrial stromal cells (nESCs) were carried out according to a previous method^[19]^. Briefly, 0.1% Type Ⅳ collagenase (Cat. #17104019, Gibco, Grand Island, NY, USA) was used to digest the endometrial tissues at 37 ℃. After filtration, the supernatant was centrifuged at 1587 *g* and the ESCs were cultured in 5% CO_2_ at 37 ℃ with DMEM/F-12 (Gibco) containing 10% fetal bovine serum (FBS; A3160901, Gibco). Lactate (10 mmol/L; Cat. # HY-B2227, MedChemExpress, NJ, USA) was used to treat cells for 24 h. Glycolysis inhibitor 2-deoxy-D-glucose (2-DG, 5 mmol/L; Cat. # HY-13966, MedChemExpress) was used to treat cells for 48 h.

### Transfection

The siRNAs were purchased from Gene-Pharma (Shanghai, China). The *LDHA* siRNA sequence was: 5′-TCGAAATGCAGACTCAAGC-3′. Briefly, siRNAs were combined with Lipofectamine 3000 (Invitrogen, Carlsbad, CA, USA) to form complexes and added to the cells. For six-well plate transfection, 5 nmol siRNA was mixed with 5 μL Lipofectamine 3000 Reagent, and the mixture was incubated for 10 min. Then, it was added to the medium. The medium was changed after 6 h. The treated cells were used in subsequent experiments.

### Quantitative RT-PCR

The treated cells were lysed by Trizol (Vazyme, Nanjing, Jiangsu, China), and 1 μg of RNA was reverse-transcribed into cDNA using cDNA synthesis kit (R211-01, Vazyme). Real-time PCR (qPCR) was performed using the SYBR Green (Vazyme) and the Applied Biosystems 7500 Real-time PCR System. The sequences of primer pairs: *LDHA* forward, 5′-TTGACCTACGTGGCTTGGAAG-3′, *LDHA* reverse, 5′-GGTAACGGAATCGGGCTGAAT-3′; *GAPDH* forward, 5′- GGAGCGAGATCCCTCCAAAAT-3′, *GAPDH* reverse, 5′- GGCTGTTGTCATACTTCTCATGG -3′.

### Wound healing assay

The treated eESCs and nESCs (5 × 10^5^) were seeded in a six-well plate. A linear gap was generated by a 200 μL pipette tip, and the picture was taken. The medium was placed with DMEM without FBS. After 24 h, the picture of the same place was taken by the microscope, and IPP software (MEDIA CYBERNETICS, USA) was used to measure the width of the scratch wound. All measurements were carried out three times^[20]^.

### Lactate detection

The lactate detection kit was purchased from Jiancheng Bioengineering Research Institute (Nanjing, Jiangsu, China). Briefly, the samples were collected, and chromogenic reagent and working buffer were added. The mixture was incubated at a chromogenic reagent for 10 min. Then, the stopping buffer was added and the absorbance was obtained at a wavelength of 530 nm.

### Transwell assay

After treatment, the eESCs and nESCs were digested and suspended, and then added into transwell chamber inserts (Millipore, Billerica, MA, USA) with matrigel (Corning Life Sciences, Tewksbury, MA, USA). Twenty-four h later, transwell chamber inserts were fixed with 4% paraformaldehyde and washed with phosphate-buffered saline (PBS) for three times. Then, the cells at the bottom were stained with Crystal Violet (Cat. #C0121, Biotime, Shanghai, China) for 4 h, and pictures were taken by the microscope. Then, the number of cells was counted by Image J in accordance with the manufacturer's instructions^[20]^.

### Cell count kit 8 (CCK8) assay

The cell viability was determined by the CCK8 assay. ESCs were placed into 96-well plates. After treatment, the CCK-8 solution was added into each well to incubate for 30 min. Absorbance was obtained by DigiScan Microplate Reader at a wavelength of 450 nm^[21]^.

### BrdU staining assay

After treatment, the cell culture medium was removed and 10 μmol/L BrdU labeling solution was added to cells. After incubating for 24 h at 37 ℃, the labeling solution was removed and the cells were washed with PBS for three times. Then, the cells were fixed by 4% paraformaldehyde. After washing, the DAPI staining solution (C1005, Beyotime, Shanghai, China) was added to stain the cell nuclear. The picture was taken under a microscope.

### Chromatin immunoprecipitation-PCR assay (ChIP-qPCR)

After treatment, cells were fixed with 4% formaldehyde and quenched with glycine (0.2 mol/L). Then, cells were sonicated, and chromatin was treated with protein A/G beads and H3K18lac antibody (1∶100; Cat. #PTM-1401RM, PTM BIO, Hangzhou, Zhejiang, China). Overnight, DNA was released from the immunoprecipitates for use in qPCR. As described previously^[18]^, the histone lactylation was enriched on the HMGB1 proximal promoter region. The primers of *HMGB1* were listed as follows: forward, TATGGCAAAAGCGGACAAGG; reverse, CTTCGCAACATCACCAATGGA.

### Western blotting analysis

The protein was lysed with the RIPA lysis buffer (P0013B, Beyotime) at 4 ℃ for 30 min. The cell debris was removed by centrifugation at 13523 *g* at 4 ℃ for 20 min. The protein concentrations were determined by the BCA Protein Assay Kit (Beyotime). Equal amounts of total protein (30 μg/lane) were loaded and separated by 10% SDS-PAGE gels. Anti-LDHA antibody (1∶1000 dilution; Cat. #3582), anti-GAPDH antibody (1∶1000 dilution; Cat. #5174), anti-c-Myc antibody (1∶1000 dilution; Cat. #18583), anti-β-tubulin antibody (1∶1000 dilution; Cat. #2128), anti-Histone H3 antibody (1∶1000 dilution; Cat. #4499), anti-HMGB1 antibody (1∶1000 dilution; Cat. #6893), anti-AKT antibody (1∶2000 dilution; Cat. #9272), anti-p-AKT antibody (1∶2000 dilution; Cat. #4060), or anti-Cyclin D antibody (1∶2000 dilution; Cat. #55506) purchased from Cell Signaling Technology (Danvers, MA, USA) were added respectively, and incubated at 4 ℃ overnight. After incubation of secondary antibodies, the membranes were washed by Tris-buffered saline with Tween 20 (0.05%) and exposed to ECL (Millipore). Images were captured using a gel imaging analysis system (Bio-Rad, Hercules, CA, USA).

### Statistical analysis

SPSS 20.0 was used for statistical analysis. The *P*-values were determined by using an unpaired student's *t*-test or Analysis of Variance (ANOVA). All data in the graphs were shown as mean ± standard deviation. *P* < 0.05 was considered statistically significant.

## Results

### The upregulated lactate, LDHA, and H3K18lac levels in ectopic endometrial tissues and primary stromal cells

**Figure 1:**
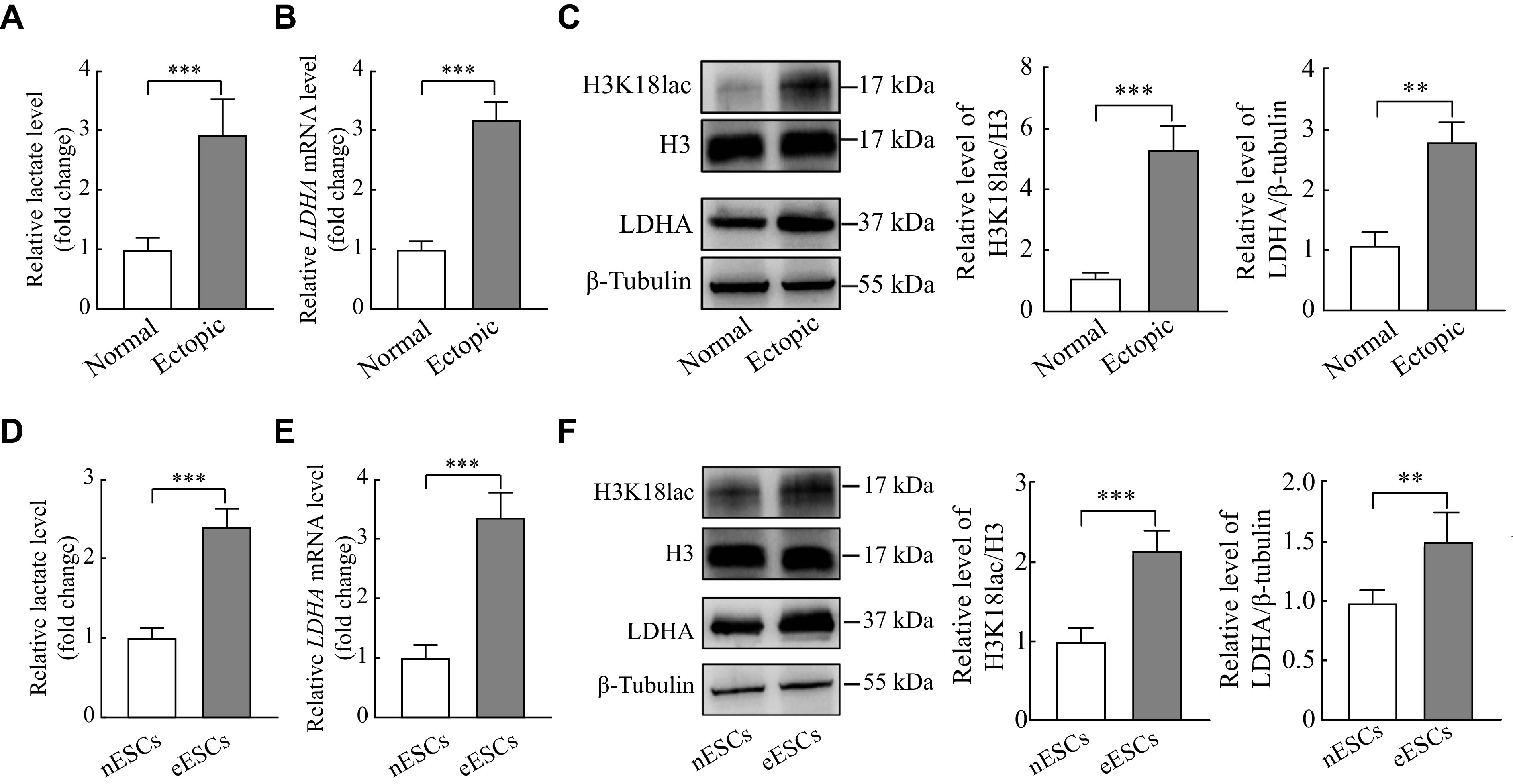
The upregulated lactate, LDHA, and H3K18lac levels in ectopic endometrial tissues and eESCs.

It was observed that the lactate levels were significantly upregulated in the ectopic endometrial tissues, compared with the normal endometrial tissues (***Fig. 1A***). Both the mRNA and protein levels of LDHA in ectopic endometrial tissues were significantly elevated, compared with the normal endometrial tissues, so as the H3K18lac protein level (***Fig. 1B*** and ***1C***). Consistent with the tissues, the lactate levels as well as LDHA and H3K18lac expression were significantly increased in the primary eESCs, compared with nESCs (***Fig. 1D***–***1F***). These results indicated higher levels of lactate, LDHA, and H3K18lac expression present in ectopic endometrial tissues and eESCs.

### Lactate promoted proliferation, migration, and invasion of primary endometrial stromal cell

**Figure 2:**
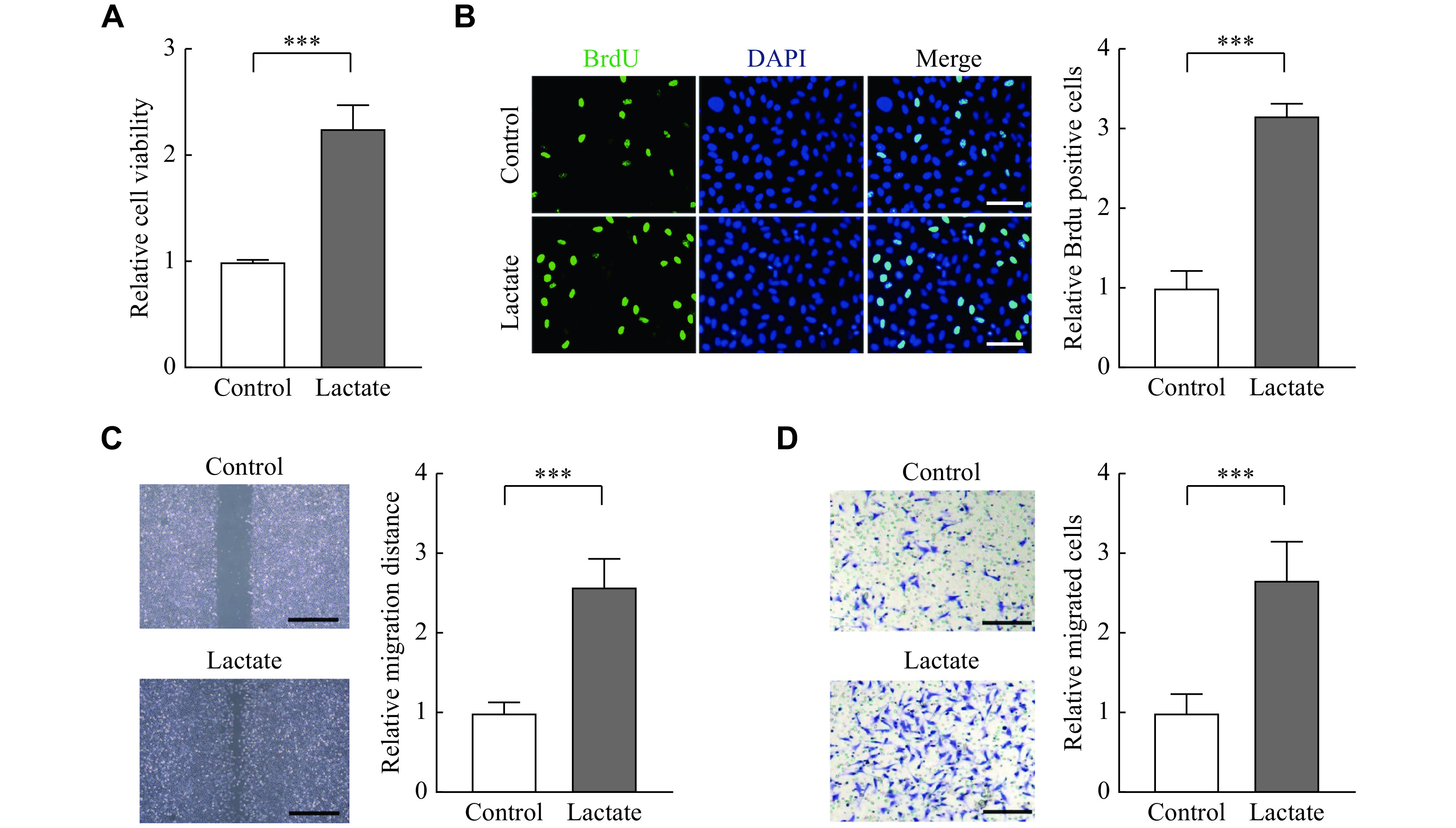
Lactate promoted cell proliferation, migration, and invasion of nESCs.

**Figure 3:**
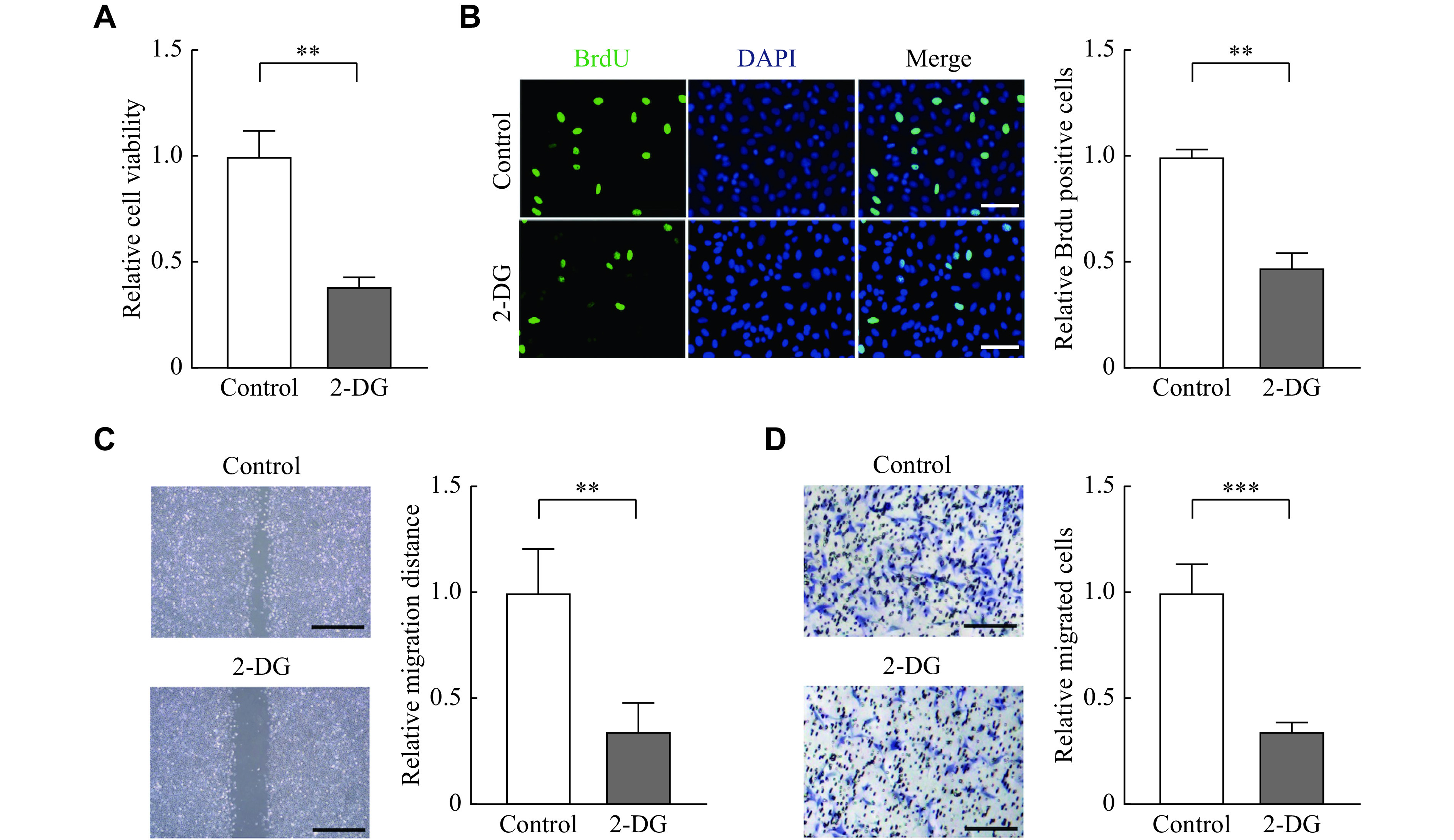
2-DG reduced proliferation, migration, and invasion of eESCs.

To investigate biological effects of lactate in the pathogenesis of endometriosis, we treated nESCs with lactate. The CCK8 assay showed that lactate could enhance cell viability (***Fig. 2A***), and the BrdU staining indicated that the proliferation of nESCs was enhanced under lactate stimulation (***Fig. 2B***). The wound healing assay and transwell assay also demonstrated that the migration and invasion ability of nESCs was improved after lactate treatment (***Fig. 2C*** and ***2D***). Then, glycolysis inhibitor 2-DG was used to block lactate in eESCs, and the results showed that 2-DG reduced the proliferation of eESCs (***Fig. 3A*** and ***3B***). It also showed that the migration and invasion ability of eESCs was blocked by 2-DG (***Fig. 3C*** and ***3D***). These results indicate that lactate may function in endometriosis by promoting cell proliferation, migration, and invasion.

### Lactate increased the level of HMGB1 through H3K18lac

**Figure 4:**
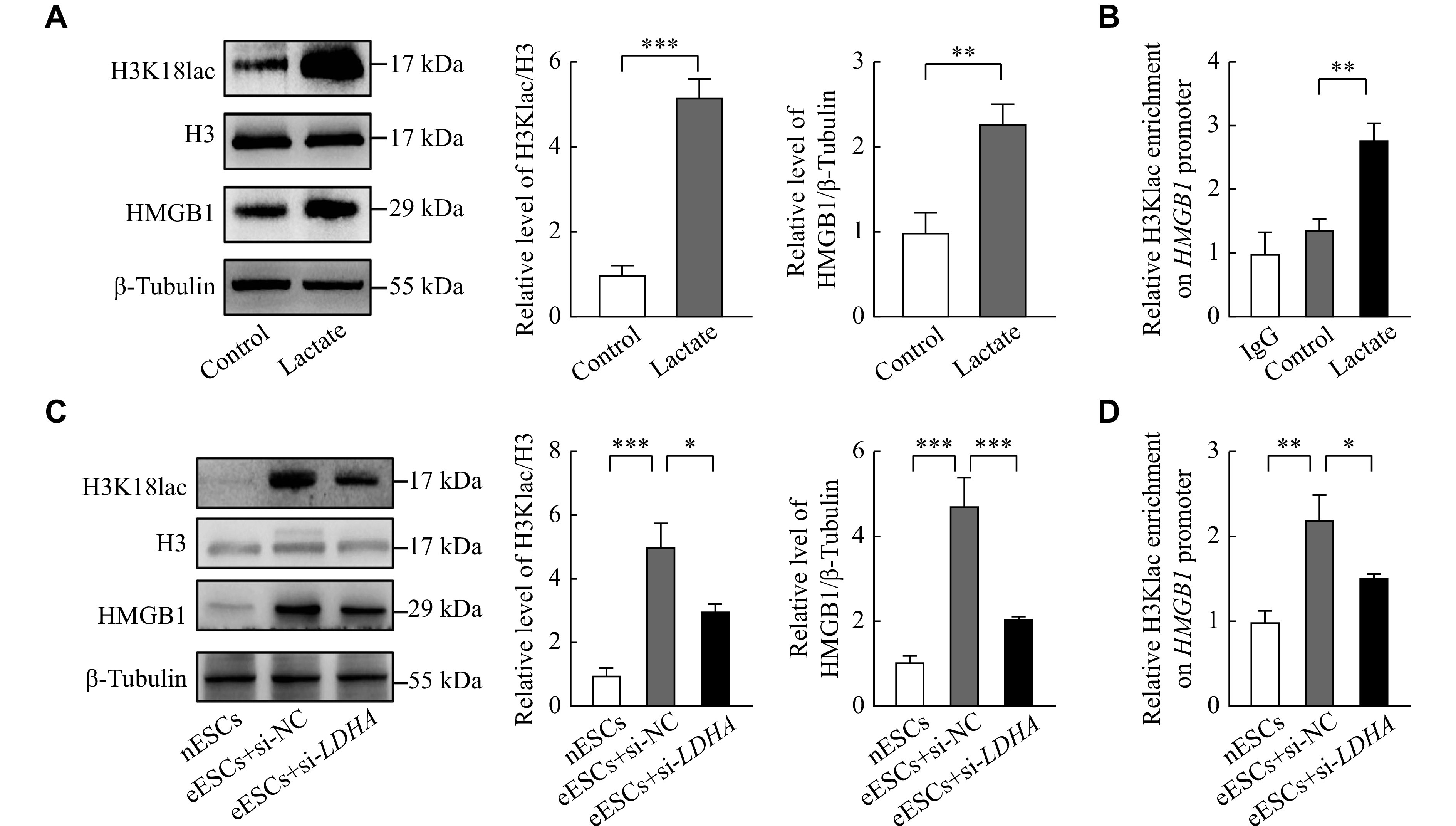
Lactate upregulated HMGB1 levels through H3K18lac.

HMGB1 is crucial in the proliferation in many cancers^[22]^. To investigate whether lactate could also promote HMGB1 expression in ESCs, lactate was used to treat nESCs. The results showed that lactate enhanced the expression of HMGB1 and H3K18lac levels (***Fig. 4A***). The ChIP-qPCR assay showed that H3K18lac was enriched in the *HMGB1* promoter region (***Fig. 4B***). Because LDHA is crucial in producing lactate in glycolysis, the *LDHA* siRNA was used to reduce the production of lactate in eESCs. The results showed that HMGB1 expression and H3K18lac levels were significantly higher in eESCs, compared with nESCs, but *LDHA* siRNA could reduce HMGB1 expression in eESCs, compared with those transfected with negative control siRNA (***Fig. 4C***). The ChIP-qPCR assay also showed that *LDHA* siRNA reduced the levels of H3K18lac enrichment in the promoter region of *HMGB1* (***Fig. 4D***). These results indicate that lactate may promote the expression of HMGB1 in endometriosis by mediating H3K18lac.

### HMGB1 knockdown reversed the role of lactate in endometrial cells

**Figure 5:**
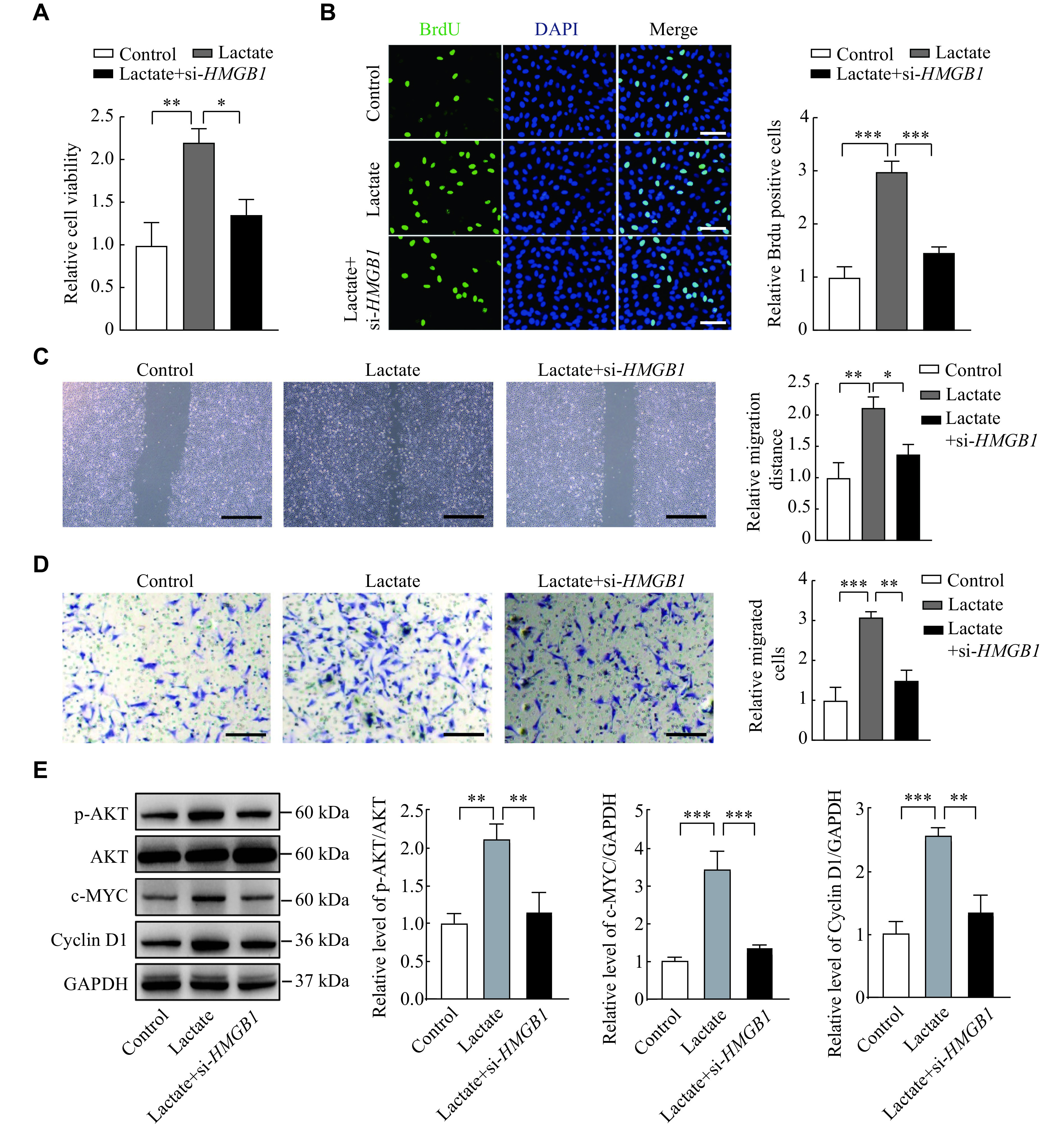
HMGB1 knockdown reversed the role of lactate in endometrial cells.

We carried out rescue experiments to investigate the lactate/HMGB1 axis in endometriosis. The CCK-8 and BrdU assays demonstrated that the knockdown of *HMGB1* could significantly reduce cell viability and proliferation of the lactate-treated cells, respectively (***Fig. 5A*** and ***5B***). Then, the wound healing and transwell assay indicated that *HMGB1* knockdown significantly reduced the migration and invasion ability of lactate-treated cells (***Fig. 5C*** and ***5D***). Western blotting assay further showed that lactate induced the phosphorylation of AKT and increased the expression of C-myc and Cyclin D1, but these changes were inhibited by the knockdown of *HMGB1* (***Fig. 5E***).

## Discussion

Endometriosis is a gynecological and estrogen-dependent disease. However, the treatment of endometriosis remains unsatisfactory^[23]^. The ability of proliferation, migration, and invasion of ESCs is similar to that in cancer cells^[24]^. Thus, to investigate the mechanism of ECSs is urgent. In the present study, we demostrated that lactate induced histone lactylation to promote endometriosis progression through upregulating the expression of HMGB1, which may provide a novel target for endometriosis treatment in the future.

Lactate can modify lactylation of histone lysine residues^[25]^. Lactylation can promote the tumor progression in many types of cancer^[26]^. In ocular melanoma, histone lactylation could promote cancer process by enhancing the expression of YTHDF2^[27]^. Previous studies indicated that ectopic ESCs produced much more lactate that induced M2 macrophage polarization by endometriosis^[28]^. Lactic acid could also protect against cell apoptosis in endometriosis^[29]^. However, the role of lactate-mediated lactylation in endometriosis remains unclear. We first indicated that the higher levels of lactate, and LDHA enhanced the H3K18lac in ectopic endometrial tissues and eESCs, and that lactate promoted cell proliferation, migration, and invasion *in vitro* in endometriosis. However, we did not check the levels of lactate and H3K18lac in eutopic endometrial tissues and eutopic endometrial stromal cells. Therefore, the lactylome analysis should be used to uncover the impact of lactylation on endometriosis in the future.

HMGB1 is a regulatory factor in many diseases, and involved in inflammation, cell death, and cell migration. In endometriosis, prostaglandin E2 increased the expression of HMGB1 and promoted the process of pyroptosis^[30]^. High plasma HMGB1 has been recognized as a biomarker for endometriosis^[31]^. Mechanistically, HMGB1 activated the PI3K/AKT/mTOR signaling pathway to increase cell proliferation and migration, promoting endothelial-mesenchymal transition in pulmonary fibrosis^[32]^. In the present study, we showed that lactate mediated H3K18lac to promote the expression of HMGB1 in endometriosis, and HMGB1 knockdown significantly decreased the cell viability, migration, and invasion of the lactate-treated nESCs; besides, lactate induced the expression of HMGB1 and increased the phosphorylation of AKT and the expression of c-MYC and Cyclin D1, all of which could be blocked by HMGB1 silencing.

**Figure 6:**
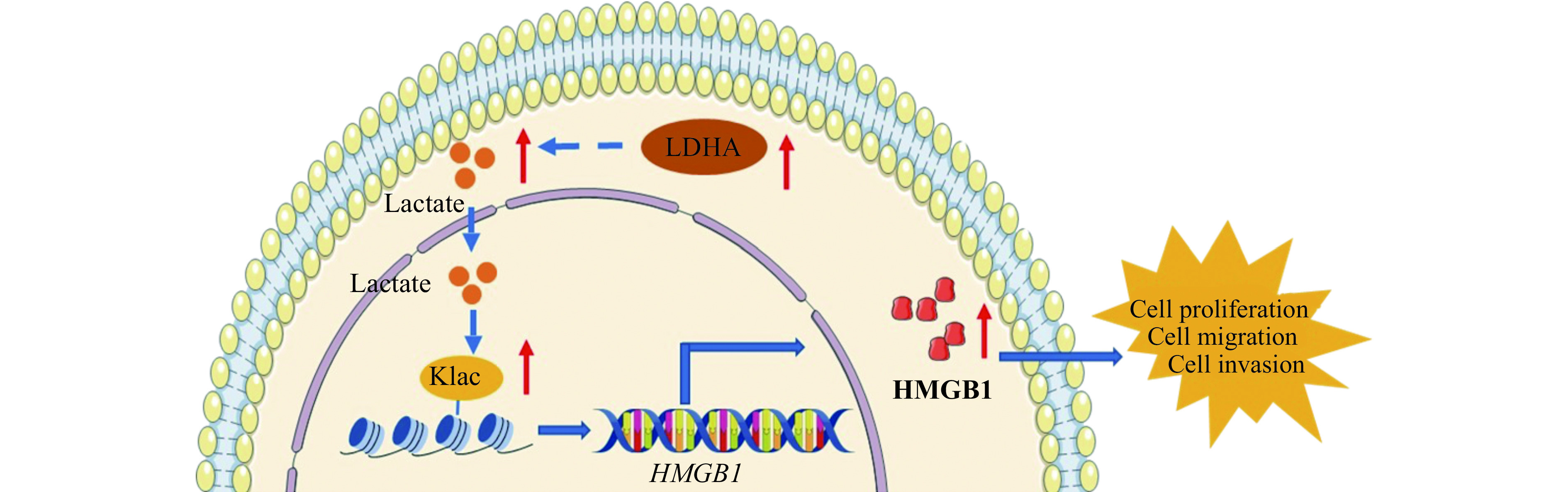
A schematic representation of how lactate promotes endometriosis progression.

In conclusion, we show that the higher expression of LDHA induces higher levels of lactate in endometriosis. The lactate may induce histone lactylation to promote endometriosis progression through upregulating the expression of HMGB1 (***Fig. 6***). In the future, we plan to use lactylome analysis to uncover more proteins that are lactylated in endometriosis. This approach has the potential to provide a novel target for the treatment of endometriosis.

## Acknowledgments

None.
